# CCDC86-BHLHE40-ATF3 axis promotes aerobic glycolysis and tumor development in glioma

**DOI:** 10.1016/j.gendis.2025.101643

**Published:** 2025-04-12

**Authors:** Jinping Liu, Dingyu Du, Yukai Huang, Jie Tian, Xuhui Wang, Longyi Chen, Feng Wang

**Affiliations:** a Department of Neurosurgery, Sichuan Provincial People's Hospital, School of Medicine, University of Electronic Science and Technology of China, Chengdu, Sichuan 610072, China; b Department of Neurosurgery, Xinhua Hospital Affiliated to Shanghai Jiao Tong University School of Medicine, Shanghai 200092, China; c Department of Medical Oncology, Cancer Center, West China Hospital, West China Medical School, Sichuan University, Sichuan 610041, China

**Keywords:** ATF3, CCDC86, ERK/MAPK signaling, Glioma, Glycolysis

## Abstract

Glioma, an aggressively malignant brain tumor with a poor prognosis, comprises nearly 50% of all primary malignant brain tumors. Despite its significance in other cancers, the role of coiled-coil domain containing 86(CCDC86) in glioma remains largely unexplored. Our study revealed a significant up-regulation of CCDC86 expression in glioma tissues, correlating notably with patient age, tumor recurrence, and pathological grade. Moreover, elevated CCDC86 level was associated with a worsened prognosis among glioma patients. Functional assays demonstrated that CCDC86 knockdown attenuated glioma cell proliferation and migration while inducing apoptosis and cell cycle arrest *in vitro* and inhibited tumorigenesis *in vivo*. Furthermore, ATF3 emerged as a downstream target gene of CCDC86, as its knockdown could counteract the oncogenic effects induced by CCDC86 overexpression in glioma cells. Mechanistically, CCDC86 promoted the transcriptional regulation of ATF3 by BHLHE40 through interaction with it, stabilizing the expression of ATF3. Additionally, our investigation unveiled a potential mechanism whereby CCDC86 activated the ERK signaling pathway through ATF3, thus influencing glycolysis to drive tumor progression. In conclusion, our study highlights the pivotal role of CCDC86 in glioma progression, suggesting its potential as a therapeutic target for the development of novel glioma treatments.

## Introduction

Glioma represents a formidable challenge in oncology, constituting nearly half of all primary malignant brain tumors.^1^ It is characterized by rapid growth, extensive infiltration, and formidable resistance to therapy. Despite current treatments combining surgical resection, radiation, and chemotherapy, their efficacy is limited and often accompanied by significant toxicity. Additionally, the genetic diversity within gliomas complicates the development of targeted therapies. Common genetic alterations in gliomas involve pivotal oncogenes such as epidermal growth factor receptor (EGFR), phosphatase and tensin homolog (PTEN), and tumor protein 53 (TP53), highlighting their critical roles in tumor progression and survival.^2^ Despite intensive research into these oncogenes as potential therapeutic targets, glioma remains largely incurable, with a median survival rate of only 15 months.^3^ Given these challenges, there is an urgent need for the development of novel treatment modalities and therapies to improve outcomes for glioma patients.

Coiled coils represent a prevalent architectural element within the proteome, discernible not only in proteins that contribute to structural integrity but also in those that modulate a spectrum of intracellular regulatory activities.^4^ Coiled coils are integral to signal transduction and serve as a molecular recognition system, endowing cells with the necessary mechanical stability and facilitating movement processes.^5^ The significance of coiled-coil-containing proteins extends to the realm of oncology, with mounting evidence indicating that their dysregulated expression is implicated in the neoplastic behavior of various cancers.^6^, ^7^, ^8^ Furthermore, coiled-coil domain-containing 109B (CCDC109B) has emerged as an oncogene and a prognostic indicator in gliomas.^9^ Coiled-coil domain-containing 86 (CCDC86), also known as CYCLON, is an approximately 80 kDa protein belonging to the coiled-coil domain-containing family. It plays pivotal roles in various cellular processes, including cell proliferation, apoptosis, and migration.^10^ Recent research has shed light on the critical involvement of CCDC86 in oncogenesis and tumor progression. Specifically, CYCLON has been identified as a driver of autonomous tumor growth, synergizing with MYC to propel the aggressive spread of lymphoma.^11^ Besides, CCDC86 has been shown to enhance the metastatic potential of nasopharyngeal carcinoma by up-regulating EGFR and activating the phosphoinositide 3-kinase (PI3K)/protein kinase B (Akt) signaling pathway.^12^ Despite its significance in other cancers, the role of CCDC86 in glioma remains unexplored.

Metabolic reprogramming emerges as a pivotal hallmark of cancer and a promising avenue for therapeutic intervention.^13^ Coiled-coil domain-containing proteins have been reported to be implicated in metabolic reprogramming. For instance, coiled-coil domain-containing 3 (CCDC3) has been shown to modulate multiple lipid metabolism pathways and alter the lipid profile through an endocrine action, thereby reprogramming lipid mechanisms.^14^ Additionally, tripartite motif containing 35 (TRIM35), which contains a coiled-coil domain, interacts with pyruvate kinase M2 (PKM2), and this domain is required for such an interaction. Importantly, the coiled-coil domain has been implicated in mediating the Warburg effect in hepatocellular carcinoma cells.^15^ Given the structural and functional similarities between CCDC86 and proteins like CCDC3 and TRIM35, we hypothesize that CCDC86 might influence metabolic reprogramming. Glycolysis, a conserved metabolic pathway, converts glucose into pyruvate, generating critical biomass intermediates such as ATP and NADH crucial for cell growth and survival, notably in glioma.^16^, ^17^, ^18^ Gliomas exhibit heightened aerobic glycolysis compared with normal brain tissue, potentially fueling their malignant progression.^17^ This metabolic shift is intricately regulated by tumor suppressors and oncogenic signaling pathways, including AKT and extracellular signal-regulated kinase (ERK), in both tumor and normal cells.^19^^,^^20^ Dysregulated expression of oncogenes and tumor suppressor genes in glioma results in alterations in the expression and activity of glycolytic transporters and metabolic enzymes. Consequently, elucidating these molecular and metabolic signatures in glioma holds promise for understanding the disease's molecular pathogenesis.

Here, we investigated the expression of CCDC86 in human glioma tissues and cell lines by analyzing our cohort and publicly available molecular databases. Then, functional experiments were performed with model systems *in vitro* and *in vivo*. We uncovered a potential oncogenic role for CCDC86 in glioma progression and identified activating transcription factor 3 (ATF3) as a downstream target gene. These results support CCDC86 as a new therapeutic target for the treatment of human glioma.

## Materials and methods

### Ethical approval

Approval for this study was obtained from the Ethics Committee of Sichuan Provincial People's Hospital (Approval No. 2024207). The research adhered to the ethical guidelines outlined in the Declaration of Helsinki.

### Bioinformatics analysis

The expression of CCDC86 was evaluated utilizing data from GSE134470, sourced from the GEO database (https://www.ncbi.nlm.nih.gov/geo/query/acc.cgi?acc=GSE134470). This dataset comprised two cases of human normal brain tissues and six cases of human glioma patient tumors. For a broader expression analysis, we utilized the GSE16011 dataset and applied the R packages ‘affy’ for data normalization and ‘limma’ for CCDC86 and ATF3 differential expression analysis. To assess the prognostic significance of CCDC86 and ATF3, we analyzed glioma samples from the Chinese Glioma Genome Atlas (CGGA) database. We downloaded the corresponding clinical information and expression data and processed the RNA sequencing data by transforming the FPKM (fragments per kilobase of transcript per million mapped reads) expression matrices of mRNAseq_693 and mRNAseq_325 using the R package ‘sva’ and its ‘combat’ function to adjust for batch effects. We also performed an expression correlation analysis using the Spearman correlation on the transformed data from the CGGA database. To understand the molecular mechanisms, we identified CCDC86-interacting proteins using the STRING database and extracted ATF3 and transcription factor regulatory relationships from the HTFTarget and humanTFDB databases. Gene Set Enrichment Analysis (GSEA) was conducted using the GSEA software (version 4.2.3) with gene sets from the MSigDB database, which includes pathways from KEGG, BIOCARTA, PID, REACTOME, WIKIPATHWAYS, and Gene Ontology. For GSEA, we used normalized expression data from glioma samples in the METABRIC database, sourced from cBioPortal, and divided samples into high and low CCDC86 expression groups based on the median expression level. The analysis was performed with 1000 permutations, considering gene sets with 15–500 genes, and normalized enrichment score (NES) > 0 indicated pathway activation in the high expression group, while NES <0 indicated pathway inhibition.

### Clinical sample collection

The collection of clinical samples for this study was conducted with strict adherence to ethical guidelines and institutional review board approval. All samples were obtained from the Sichuan Provincial People's Hospital. Patients diagnosed with glioma were considered for inclusion in the study. Inclusion criteria specified that patients must have undergone surgical resection of the tumor. Exclusion criteria were applied to ensure the study's focus on glioma, excluding patients with a history of other malignancies or those receiving neoadjuvant therapy that could affect tumor biology. Informed consent was obtained from all patients.

### Immunohistochemistry staining

A glioma tissue microarray, consisting of 150 tumor tissues and 40 normal tissues, was provided by Shanghai Yibeirui Biomedical Science and Technology Co., Ltd., China. Tissue sections were subjected to overnight incubation with primary antibodies at 4 °C, followed by a 1-h incubation with specific secondary antibodies at room temperature. Subsequent steps included treatment with horseradish peroxidase (HRP)-labeled streptavidin for 1 h, staining with diaminobenzidine for 5 min, and counterstaining with hematoxylin (Gene Tech, China). Evaluation of immunohistochemistry staining results was performed blindly by two experienced pathologists. Details of the antibodies used are listed in Table S1.

### Cell culture

Human embryonic brain cells were cultured in Dulbecco's modified Eagle medium (DMEM) supplemented with 10% fetal bovine serum. Glioma cell lines U251, U373, SHG-44, and U87 were cultured in DMEM supplemented with 10% fetal bovine serum and 1% penicillin/streptomycin. All cells were maintained at 37 °C in a humidified atmosphere with 5% CO_2_.

### Establishment of indicated gene overexpression and knockdown plasmids

Shanghai Yibeirui Biomedical Science and Technology Co., Ltd. designed lentiviral shRNAs to target CCDC86 (sequences: AGCGTCCAAGAAGTTGAATAA, GGAGAATGAGCGGAAGGCAGA, and GCGGGCAAAGAAGAAGCAGCT) and ATF3 (sequences: CCTGAAGAAGATGAAAGGAAA, GACTCCAGAAGATGAGAGAAA, and GTCGGAGAAGCTGGAAAGTGT). To facilitate CCDC86 or basic helix-loop-helix family member E40 (BHLHE40) overexpression, the gene sequence underwent optimization based on codon usage preferences, followed by synthesis and cloning into T4 DNA Ligase (EL0016, Fermentas). The efficacy of knockdown and overexpression was validated through western blotting or real-time quantitative reverse transcription PCR (qRT-PCR).

### qRT-PCR

In qRT-PCR, cellular RNA was extracted and converted into complementary DNA (cDNA), which was subsequently diluted for analysis using SYBR® Premix Ex Taq™ II (from TaKaRa, Thermo Fisher Scientific). GAPDH was utilized as an internal reference to normalize gene expression levels, calculated via the 2^−ΔΔCt^ method. The experiment was repeated three times to ensure reproducibility and accuracy. Primer sequences are available in Table S2.

### Western blotting

For western blotting experiments, protein extraction was carried out, and protein concentration was determined using Beyotime's BCA protein assay kit. Equal amounts of protein were loaded onto a 10% SDS-PAGE gel and transferred onto Millipore's PVDF membranes. Blocking with 5% skimmed milk in Tris-buffered saline containing 0.1% Tween 20 (TBST) preceded overnight incubation with primary antibodies at 4 °C. Following three TBST washes, membranes were treated with secondary HRP antibodies at room temperature for 1 h. After three additional TBST washes, appropriate HRP-conjugated secondary antibodies were applied, and enhanced chemiluminescence facilitated immunoblot analysis. GAPDH served as a normalization control, and densitometry analysis was conducted using Image J software. Details regarding the antibodies utilized are shown in Table S1.

### Celigo cell counting experiment

Cells transfected with lentivirus (SHG-44 and U251) were fixed and stained with fluorescent dye. Imaging was conducted using the Celigo Imaging Cytometer, with Celigo software automating cell identification and counting. Data analysis ensued to determine cellular proliferation rates. The experiment was repeated three times to ensure reproducibility and accuracy.

### CCK-8 detection

For assessing glioma cell growth rates, a CCK-8 assay was performed. Cell samples, collected 48 h post-transfection, were plated into 96-well plates (2 × 10^3^ cells per well). Following incubation with CCK-8 solution and medium (200 μL) at 37 °C for 2 h, optical density was measured at 450 nm using an absorbance microplate reader. Absorbance readings correlated with viable cell numbers over the subsequent five days. The experiment was repeated three times to ensure reproducibility and accuracy.

### Wound-healing assay

After a 48-h transfection period, cells (100 μL) were seeded into 24-well plates at a density of 5 × 10^5^ cells/mL. Scratch wounds were created, and cells were cultured in a medium containing 10 g/L bovine serum albumin and 1% fetal bovine serum. Migration distance was quantified under a microscope, with subsequent observation of relative migration towards the wound area after maintaining cells in medium with 10% fetal bovine serum. The experiment was repeated three times to ensure reproducibility and accuracy.

### Transwell assay

After a 24-h period of serum deprivation, cells underwent two washes with phosphate buffer saline and were then suspended in serum-free Opti-MEM at a density of 5 × 10^4^ cells/mL. These cells were subsequently seeded into the upper chambers of 24-well transwell plates (8 μm pore size) with 100 μL per well in triplicates. The lower chambers were filled with 600 μL of DMEM or minimum essential medium (MEM) for culture. Following a 48-h incubation period, cells were fixed with 4% paraformaldehyde for 10 min, washed thrice with phosphate buffer saline, and stained with crystal violet for another 10 min. Cell migration was assessed under a microscope, with counts conducted in five randomly selected fields of view to quantify cells that traversed the membrane. The experiment was repeated three times to ensure reproducibility and accuracy.

### Flow cytometry detection for apoptosis and cell cycle

Post-lentiviral transfection, 5 × 10^5^ SHG-44 and U251 cells were trypsinized, washed with phosphate buffer saline, and centrifuged at 1300 rpm for 5 min. Following a single wash with 1 × binding buffer, cells were resuspended in 1 × staining buffer. A 2 mL/well cell suspension was incubated with 10 μL of the annexin V-APC apoptosis detection kit II and 0.1% Triton X-100 in the dark at room temperature for 15 min. Flow cytometry analysis (Millipore, Guava easyCyte HT) was employed to evaluate cell apoptosis and determine the percentage distribution of cells across different cell cycle phases (G1, S, and G2). The experiment was repeated three times to ensure reproducibility and accuracy.

### PrimeView human gene expression array

Total RNA was meticulously extracted from the samples following well-established protocols. The quality and integrity of extracted RNA were subjected to stringent evaluation using the Nanodrop 2000 spectrophotometer from Thermo Fisher Scientific (Waltham, Massachusetts, USA) and the Agilent 2100 Bioanalyzer equipped with the RNA 6000 Nano Kit, provided by Agilent, Santa Clara, California, USA. The Affymetrix GeneChip PrimeView platform was then engaged for comprehensive RNA sequencing, with all procedures being conducted in strict accordance with the manufacturer's detailed guidelines. The generated data underwent scanning via the Affymetrix Scanner 3000, ensuring high-quality data acquisition. For the statistical analysis of the gene expression data, we applied Welch's *t*-test, complemented by the Benjamini-Hochberg false discovery rate (FDR) correction. Genes with |fold change|) ≥ 1.3 and FDR < 0.05 were considered significant.

### Co-immunoprecipitation assay

Cells were lysed in RIPA buffer supplemented with protease and phosphatase inhibitors. The lysates were then subjected to centrifugation to remove debris. The supernatant was incubated with an anti-BHLHE40 antibody at 4 °C overnight. Protein A/G magnetic beads were added, and the mixture was incubated for an additional 24 h. The beads were washed extensively with RIPA buffer to remove non-specifically bound proteins. The immunoprecipitated complexes were eluted and analyzed by western blotting using an anti-CCDC86 antibody to confirm the interaction.

### Nuclear fractionation assay

SHG-44 and U251 cells were transfected with shCCDC86 or shCtrl. After 48 h, cells were harvested and lysed in a hypotonic buffer. Nuclei were isolated by centrifugation, and the supernatant containing the cytoplasmic fraction was collected. The nuclear pellet was resuspended in nuclear lysis buffer. Both fractions were subjected to western blotting analysis using antibodies against BHLHE40, histone H3 (as a nuclear marker), and GAPDH (as a cytoplasm marker) to determine the distribution of BHLHE40.

### Chromatin immunoprecipitation assay

SHG-44 and U251 cells were fixed with 1% formaldehyde to cross-link proteins to DNA. The cross-linked chromatin was sonicated to generate DNA fragments of approximately 200–500 bp. After centrifugation, the supernatant was immunoprecipitated with an anti-BHLHE40 antibody or a non-specific IgG control. The immunoprecipitated DNA was reverse-cross-linked and purified. Enrichment of ATF3 promoter sequences was assessed by qRT-PCR, using primers specific to the ATF3 promoter region. Primer sequences are available in Table S2.

### Construction of xenograft tumor model

Animal xenograft model studies adhered to institutional guidelines. Lentivirus-transfected U251 cells (1 × 10^7^ cells/mice) were subcutaneously injected into the right armpit region of four-week-old female BALB/c nude mice, randomized into four groups (*n* = 4 mice/group). Tumor diameters were measured every five days using a caliper to calculate tumor volume. On day 28 post-injection, mice were euthanized, and tumors were excised and weighed. All animal procedures were ethically approved by the Ethics Committee of Sichuan Provincial People's Hospital (Approval No. 2024207).

### ATP content, glucose uptake, and lactate production

ATP content, glucose uptake, and lactate production were determined using the ATP content kit (Solarbio, #BC0300), glucose uptake kit (Solarbio, #BC2500), and lactate assay kit (Solarbio, #BC2230), respectively, as per their manufacturer's instructions.

### Statistical analysis

The data was presented as mean ± standard deviation and analyzed using GraphPad Prism 8.0 software. Statistical significance between groups was assessed using a student's *t*-test or one-way ANOVA test. The correlation between CCDC86 expression and clinical characteristics in patients with glioma was determined through Mann–Whitney *U* analysis and Spearman rank correlation analysis. Kaplan–Meier survival analysis was conducted to evaluate the association between CCDC86 expression and patient survival outcomes. *p* values < 0.05 were deemed statistically significant.

## Results

### CCDC86 expression as a prognostic marker for glioma

**Figure 1:**
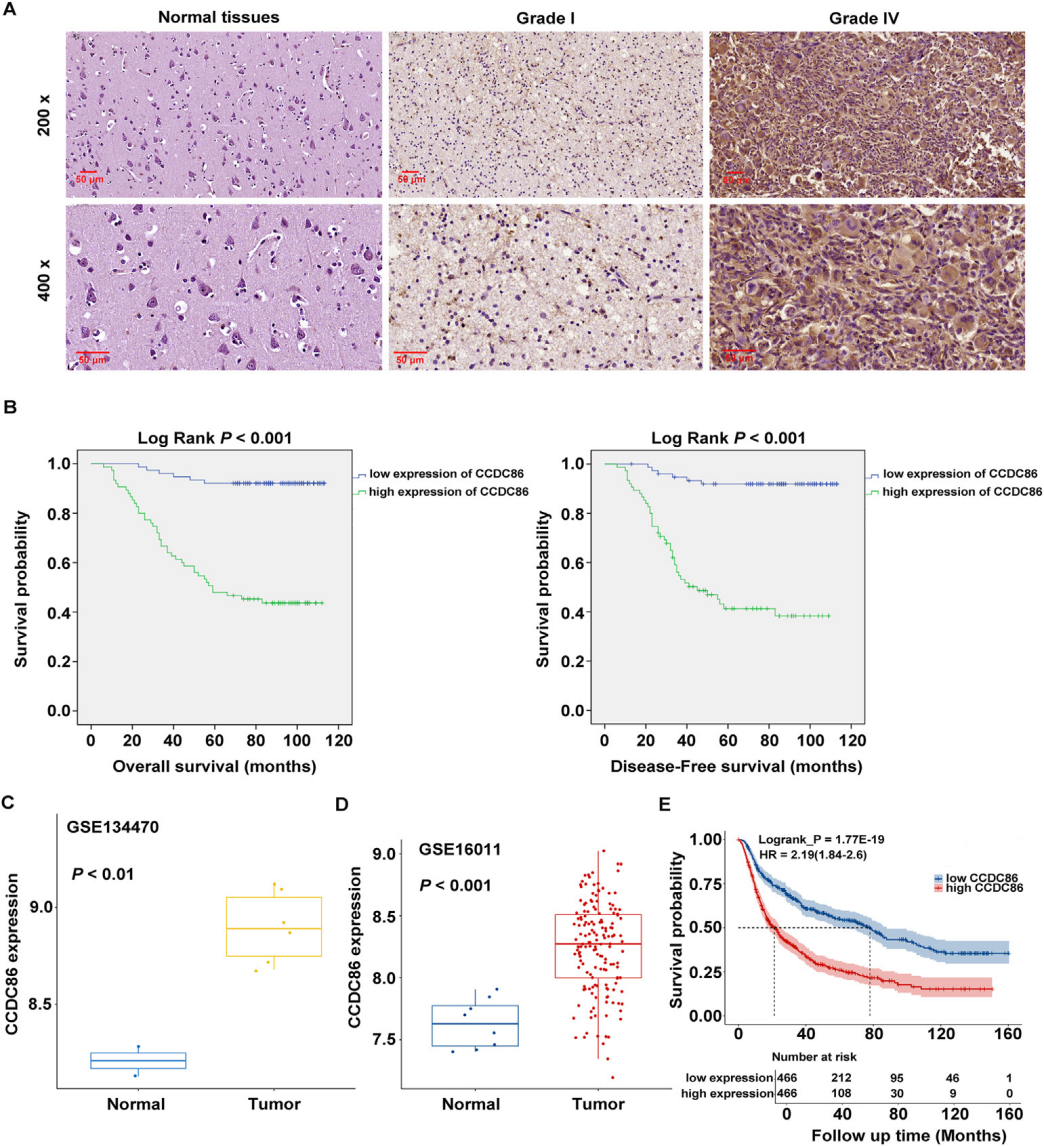
CCDC86 expression as a prognostic marker for glioma. **(A)** Immunohistochemical staining of CCDC86 in a cohort of 150 glioma tissue samples and 40 normal tissue samples. **(B)** The Kaplan–Meier survival curves depicting overall and disease-free survival in glioma patients with high/low CCDC86 expression. **(C)** Differential expression analysis of CCDC86 in glioma and normal tissues sourced from the GEO dataset GSE134470. **(D)** Differential expression analysis of CCDC86 in glioma and normal tissues sourced from the GEO dataset GSE16011. **(E)** The log-rank test results showed a significant association between high CCDC86 expression and poor overall survival in glioma patients. CCDC86, coiled-coil domain-containing 86.

In this study, we employed a tissue microarray consisting of 150 glioma tissue samples and 40 normal tissue samples for immunohistochemical staining analysis. The findings unveiled a notable up-regulation of CCDC86 in glioma tissues (Fig. 1A and Table 1). This up-regulation was further scrutinized through Mann–Whitney U and Spearman rank correlation analyses, revealing a positive correlation of CCDC86 expression with patients' age, tumor recurrence, and pathological grade (Table 2, Table 3). More importantly, Kaplan–Meier survival analysis unveiled a substantial association of CCDC86 expression with overall survival and disease-free survival in glioma patients (Fig. 1B), highlighting its potential as a prognostic marker for this disease. To bolster our findings, we analyzed CCDC86 expression in two normal human brain tissue samples and six human glioma samples sourced from the GEO database (accession number GEO: GSE134470). Differential expression analysis, using the raw expression data of individual samples, showed a significant disparity between glioma and normal tissues (log_2_foldchange = 0.6880; *p* < 0.01) (Fig. 1C). Additionally, glioma and normal tissue samples from the GSE16011 dataset were selected for further analysis. The analysis confirmed that CCDC86 was significantly overexpressed in gliomas compared with normal tissues (log_2_foldchange = 0.615; *p* < 0.001) (Fig. 1D). By taking the median expression value of CCDC86 across all glioma samples as a cutoff, the samples were divided into high and low expression groups for CCDC86. We then used the log-rank test to examine the differences in overall survival between the two groups. The results indicated that high expression of CCDC86 in gliomas was associated with poor overall survival (Logrank_P = 1.77E-19; hazard ratio = 2.19 (1.84–2.6) (Fig. 1E). Taken together, these data provide evidence for the up-regulation of CCDC86 in glioma and its association with patient survival, suggesting its potential as a prognostic marker in glioma.

**Table 1:** Expression patterns in glioma tissues and normal tissues revealed in immunohistochemistry analysis.

CCDC86 expression	Tumor tissue		Normal tissue		*p* value
	Cases	Percentage	Cases	Percentage	
Low	75	50.0%	37	92.5%	*p* < 0.001
High	75	50.0%	3	7.5%

**Table 2:** Relationship between CCDC86 expression and tumor characteristics in patients with glioma.

Features	No. of patients	CCDC86 expression		*p* value
		low	high	
All patients	150	75	75	
Age				0.001
≤41 years	78	49	29	
>41 years	72	26	46	
Gender				0.171
Male	98	45	53	
Female	52	30	22	
Tumor recurrence				*p* < 0.001
No	74	59	15	
Yes	76	16	60	
Grade				*p* < 0.001
I	23	23	0	
II	67	44	23	
III	46	7	39	
IV	14	1	13

**Table 3:** Relationship between CCDC86 expression and tumor characteristics in patients with glioma.

		CCDC86
Grade	Spearman correlation	0.638
	Signification (double-tailed)	*p* < 0.001
	N	150
Tumor recurrence	Spearman correlation	0.587
	Signification (double-tailed)	*p* < 0.001
	N	150
Age (years)	Spearman correlation	0.267
	Signification (double-tailed)	0.001
	N	150

### CCDC86 knockdown suppresses viability and migration and enhances apoptosis in glioma cells

**Figure 2:**
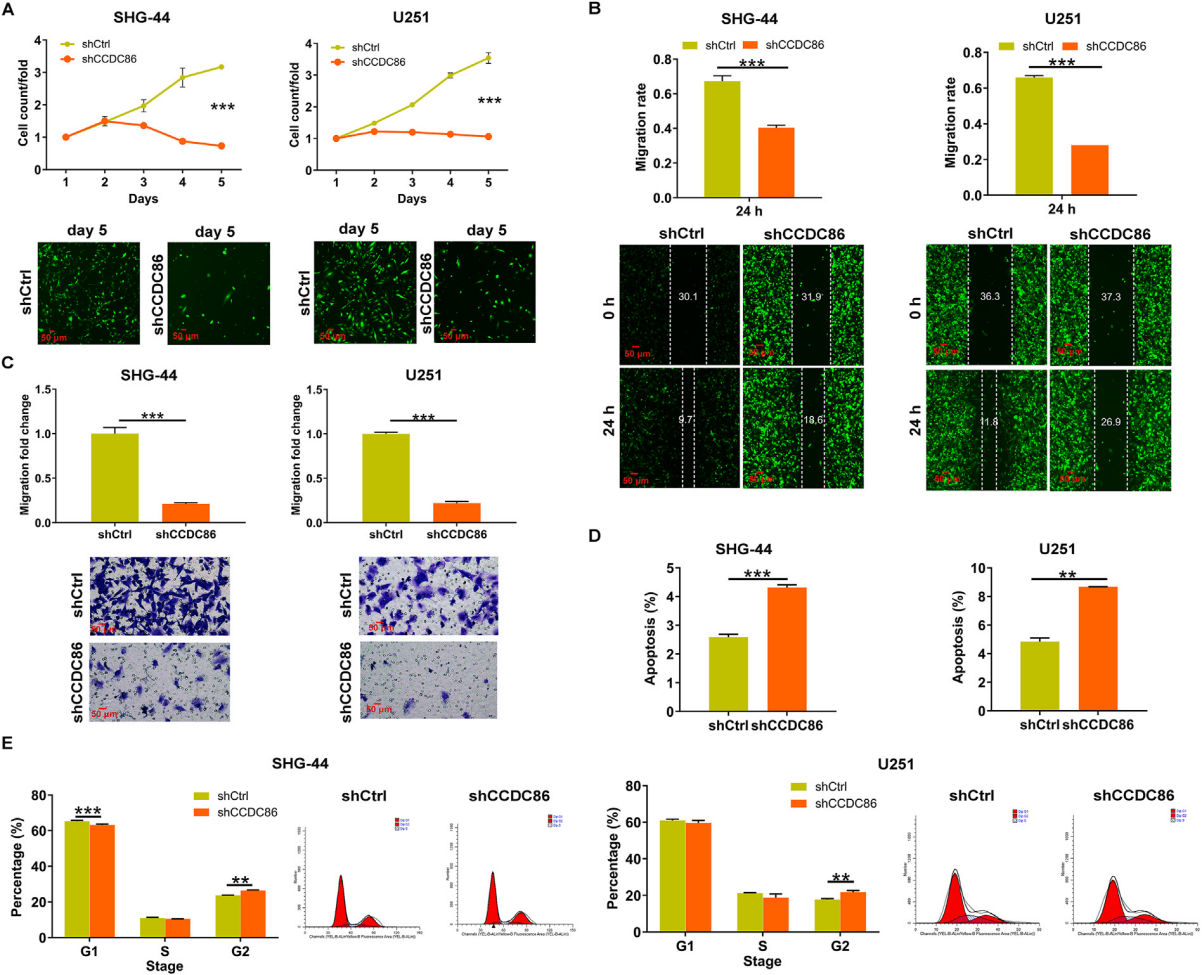
CCDC86 knockdown suppresses viability and migration and enhances apoptosis in glioma cells. **(A)** Celigo cell counting assay was utilized to assess the impact of CCDC86 knockdown on the proliferation of SHG-44 and U251 cells. Magnification: 200 × . **(B, C)** Wound-healing assay (B) and transwell analysis (C) were employed to evaluate the influence of CCDC86 knockdown on the migration of SHG-44 and U251 cells. The numbers on the healing assay images refer to the length of the wound area that has healed at 24 h post-scratch. Magnification: 200 × . **(D, E)** Flow cytometry analysis was conducted to visualize the effects of CCDC86 knockdown on apoptosis (D) and cell cycle progression (E) in SHG-44 and U251 cells. ∗∗*p* < 0.01 and ∗∗∗*p* < 0.001. CCDC86, coiled-coil domain-containing 86.

To further explore CCDC86's functional impact in glioma, we utilized three shRNAs (shCCDC86-1, shCCDC86-2, and shCCDC86-3) to target CCDC86, thereby suppressing its expression in U251 cells, which exhibited the highest endogenous CCDC86 levels (Fig. S1A, B). Among these, shCCDC86-1 displayed optimal transfection efficiency and was thus chosen for subsequent investigation (Fig. S1B). Following this selection, we conducted qRT-PCR and western blotting experiments to validate the successful knockdown of CCDC86 in SHG-44 and U251 cells with relatively higher endogenous CCDC86 levels (Fig. S1C). Functional assays were then performed to elucidate CCDC86's roles in glioma. Our findings revealed that CCDC86 knockdown significantly impeded cell viability, as evidenced by Celigo cell counting experiments (Fig. 2A). Moreover, wound-healing assays and transwell analysis demonstrated impaired cell migration in CCDC86-silenced glioma cells (Fig. 2B and C). Additionally, flow cytometry experiments indicated an enhanced propensity for apoptosis following CCDC86 down-regulation (Fig. 2D), along with cell cycle arrest at the G2 phase (Fig. 2E). Overall, our study provides compelling evidence supporting the pivotal role of CCDC86 in malignant processes within glioma cells.

### Exploring the downstream mechanism of CCDC86 regulating the progression of glioma

**Figure 3:**
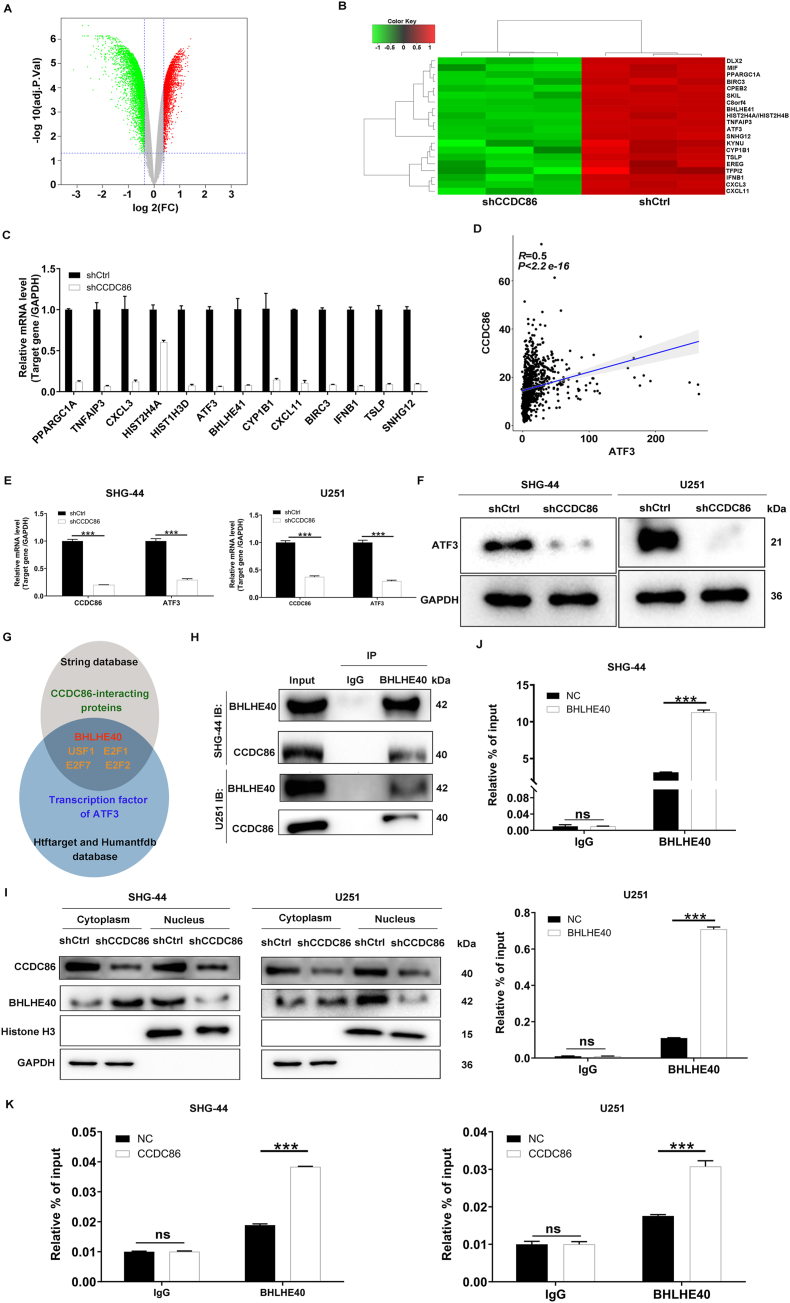
Exploring the downstream mechanism of CCDC86 regulating the progression of glioma. **(A)** The volcano plot depicting the differential expression of genes in SHG-44 cells following CCDC86 knockdown, as revealed by GeneChip PrimeView Human PathArray™ analysis. **(B)** The heat map illustrating the top 20 significantly down-regulated genes in SHG-44 cells treated with shCCDC86. **(C)** Quantitative reverse transcription PCR validation of the top 13 down-regulated genes post-CCDC86 knockdown. **(D)** The correlation between CCDC86 and ATF3 based on the glioma samples from the CGGA database. **(E, F)** Validation of ATF3 through quantitative reverse transcription PCR (E) and western blotting (F) experiments in SHG-44 cells and U251 cells with CCDC86 knockdown. **(G)** Flow chart of downstream molecular relationship construction. **(H)** Co-immunoprecipitation experiments confirmed the endogenous interaction between CCDC86 and BHLHE40. **(I)** The levels of BHLHE40 were detected in the nucleus and cytoplasm of CCDC86-depleted SHG-44 and U251 cells. **(J)** Chromatin immunoprecipitation experiments were performed to detect the binding of ATF3 promoter to BHLHE40 in BHLHE40-overexpressed SHG-44 and U251 cells. **(K)** Chromatin immunoprecipitation experiments were performed to detect the binding of ATF3 promoter to BHLHE40 in CCDC86-overexpressed SHG-44 and U251 cells. ∗∗∗*p* < 0.001. CCDC86, coiled-coil domain-containing 86; ATF3, activating transcription factor 3; BHLHE40, basic helix-loop-helix family member E40; ns, not significant.

To unravel the downstream mechanisms through which CCDC86 regulated glioma development, a GeneChip PrimeView Human PathArray™ analysis was conducted on SHG-44 cells transfected with shCtrl or shCCDC86, revealing differentially expressed genes. The analysis identified 1687 up-regulated and 2643 down-regulated genes in shCCDC86-transfected cells, with a significance threshold of |fold change| ≥ 1.3 and FDR <0.05 (Fig. 3A). The top 20 significantly down-regulated genes were highlighted (Fig. 3B). qRT-PCR validation of the top 13 down-regulated genes after CCDC86 knockdown confirmed their significant down-regulation (Fig. 3C). Additionally, an analysis of glioma samples from the CGGA database was performed to assess the correlation between CCDC86 and these 13 genes. Notably, IFNB1 was not identified in the CGGA database. CCDC86 was found to be positively correlated with ATF3, TNFAIP3, CXCL11, SNHG12, BIRC3, CXCL3, TSLP, and CYP1B1, and negatively with HIST1H3D, HIST2H4A, BHLHE4, and PPARGC1A, with ATF3 showing the highest correlation coefficient as a downstream gene (*r* = 0.5; Fig. 3D; Fig. S2A). In SHG-44 and U251 cells with CCDC86 knockdown, qRT-PCR and western blotting analysis of ATF3 mRNA and protein levels showed significant down-regulation upon CCDC86 knockdown (Fig. 3E, F). Further analysis of ATF3 expression in glioma and normal tissue samples from the GEO database (GSE16011) indicated higher expression in glioma (*P*_adj_ = 0.00578; Fig. S2B). A log-rank test comparing high and low ATF3 expression groups within glioma samples revealed that high ATF3 expression was associated with poorer overall survival (Logrank_P = 8.76E-19; hazard ratio = 2.15 (1.81–2.56) (Fig. S2C). ATF3 was also found to be up-regulated in glioma cells compared with normal human embryonic brain cells (Fig. S2D). Thus, ATF3 was identified as a target gene of CCDC86.

To explore the molecular mechanisms by which CCDC86 regulated ATF3, the interacting proteins of CCDC86 and their corresponding interaction scores were obtained using the STRING database. Additionally, ATF3 and its transcription factors were retrieved from the HTFTarget and humanTFDB databases. The intersection of these results yielded transcription factors such as BHLHE40, USF1, E2F1, E2F7, and E2F2. Among them, BHLHE40 had the highest interaction score with CCDC86 (combined_score = 283; Fig. 3G), thus, we hypothesized that CCDC86 might promote the transcriptional regulation of ATF3 by BHLHE40 through interaction with it. To address this hypothesis, the co-immunoprecipitation experiment was conducted to verify the protein interaction between BHLHE40 and CCDC86, where CCDC86 was detected in the protein complex after BHLHE40 was immunoprecipitated (Fig. 3H). Additionally, nuclear fractionation assays in CCDC86 knockdown SHG-44 and U251 cells indicated a decrease of BHLHE40 levels in the nucleus (Fig. 3I). Furthermore, chromatin immunoprecipitation assays demonstrated the enhanced enrichment of ATF3 promoter in BHLHE40-precipitate DNA complexes of BHLHE40-overexpressed SHG-44 and U251 cells relative to the NC groups (Fig. 3J; Fig. S3A). To further confirm that CCDC86 participated in the regulation of ATF3 by BHLHE40, a chromatin immunoprecipitation experiment was carried out in SHG-44 and U251 cells with overexpressed CCDC86 (Fig. S3B), where it was shown that overexpression of CCDC86 led to increased enrichment of ATF3 promoter in BHLHE40-associated DNA complexes (Fig. 3K). In summary, CCDC86 promotes the transcriptional regulation of ATF3 by BHLHE40 through interaction with it, stabilizing the expression of ATF3.

### CCDC86 regulates glioma development through ATF3 *in vitro* and *in vivo*

**Figure 4:**
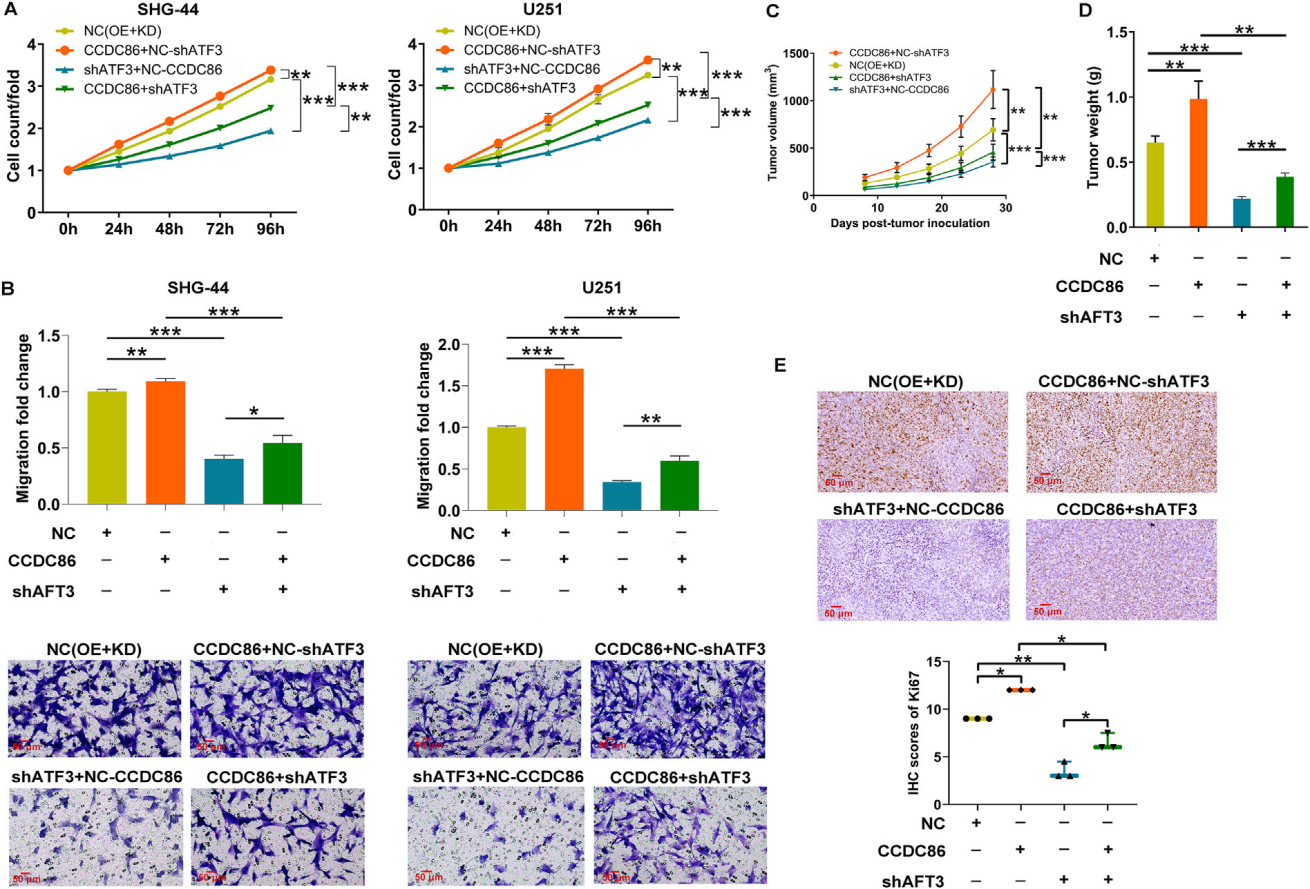
CCDC86/ATF3 regulates glioma development *in vitro* and *in vivo*. **(A, B)** Functional experiments demonstrated the effects of CCDC86 overexpression and ATF3 knockdown on SHG-44 and U251 cell proliferation (A) and migration (B). Magnification: 200 ×. **(C, D)***In vivo* tumor growth assay depicted the role of CCDC86 and ATF3 in promoting tumor growth in nude mice, including tumor volume (C) and weight (D). **(E)** Immunohistochemical staining revealed the changes in Ki67 expression in tumor tissues with CCDC86 overexpression, ATF3 knockdown, and combined CCDC86 overexpression and ATF3 knockdown. Quantification of immunohistochemical staining scores for Ki67. Magnification: 200 ×. NC (OE + KD): control; CCDC86+NC-shATF3: CCDC86 overexpression; shATF3+NC-CCDC86: ATF3 down-regulation; CCDC86+shATF3: CCDC86 overexpression and ATF3 down-regulation. ∗*p* < 0.05, ∗∗*p* < 0.01, and ∗∗∗*p* < 0.001. CCDC86, coiled-coil domain-containing 86; ATF3, activating transcription factor 3.

In this section, this study aimed to assess the roles of CCDC86 and ATF3 in glioma development *in vitro* and *in vivo*. We established cell models of SHG-44 and U251 cells transfected with different lentiviruses to knock down ATF3, overexpress CCDC86, or both. Knockdown and overexpression efficiencies were validated (Fig. S4A, B), and subsequent cell functional experiments demonstrated that CCDC86 overexpression promoted glioma cell proliferation and migration, while ATF3 down-regulation inhibited these phenotypes, even reversing the pro-cancer effects of CCDC86 overexpression (Fig. 4A, B). To investigate the roles of CCDC86/ATF3 in tumor growth, we conducted *in vivo* animal experiments. Subcutaneous injection of SHG-44 cells, either with ATF3 knockdown, CCDC86 overexpression, or both ATF3 knockdown and CCDC86 overexpression, was performed on 4-week-old nude mice. Tumor growth was monitored, revealing that CCDC86 played a crucial role in promoting tumor cell growth, evidenced by an increase in both tumor volume and weight upon its overexpression. Additionally, ATF3 knockdown effectively inhibited the oncogenic effects of CCDC86 overexpression (Fig. 4C, D). Immunohistochemical staining of tumor tissues revealed a significant increase in Ki67 expression in the CCDC86 overexpression group, whereas ATF3 knockdown led to a decrease in Ki67 expression. Furthermore, the group with both CCDC86 overexpression and ATF3 knockdown demonstrated a reduction in Ki67 expression compared with the group with CCDC86 overexpression alone (Fig. 4E). These findings collectively suggest that CCDC86 and ATF3 are pivotal in modulating glioma growth.

### The CCDC86-ATF3 axis controls the ERK/MAPK pathway and glycolytic metabolism

**Figure 5:**
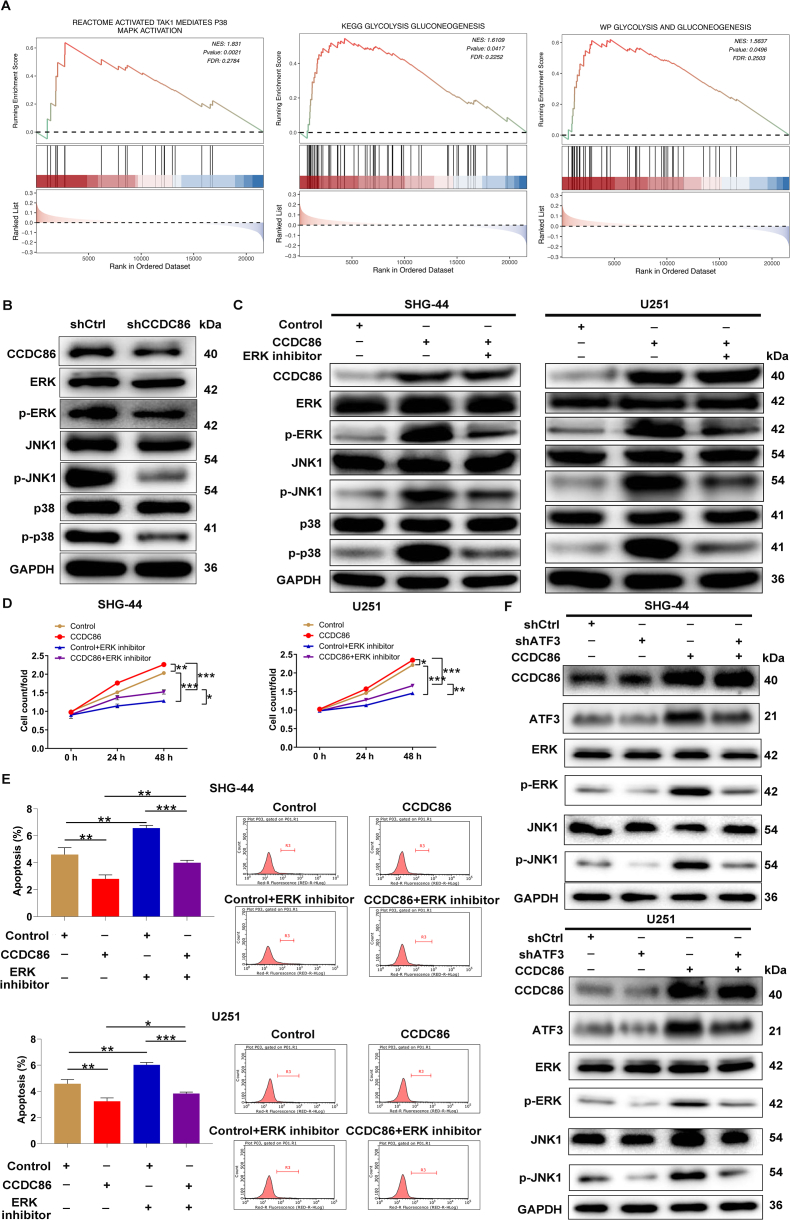
The CCDC86-ATF3 regulates the ERK/MAPK pathway. **(A)** Gene set enrichment analysis (GSEA) was performed on glioma samples from the METABRIC database to investigate the downstream pathways influenced by the CCDC86. **(B)** Effects of CCDC86 knockdown on total and phosphorylated ERK, JNK1, and p38 levels in U251 cells. **(C)** Effects of ERK inhibitor on total and phosphorylated ERK, JNK1, and p38 levels in CCDC86-overexpressed SHG-44 and U251 cells. **(D, E)** Influences of ERK inhibitor on the cell proliferation (D) and apoptosis (E) of CCDC86-overexpressed SHG-44 and U251. **(F)** Assessment of ERK and JNK1 levels and their phosphorylation status, as well as CCDC86 and ATF3 levels in SHG-44 and U251 cells with ATF3 knockdown, CCDC86 overexpression, and combined CCDC86 overexpression and ATF3 knockdown. NC (OE + KD): control; CCDC86+NC-shATF3: CCDC86 overexpression; shATF3+NC-CCDC86: ATF3 down-regulation; CCDC86+shATF3: CCDC86 overexpression and ATF3 down-regulation. ∗*p* < 0.05, ∗∗*p* < 0.01, and ∗∗∗*p* < 0.001. CCDC86, coiled-coil domain-containing 86; ATF3, activating transcription factor 3; ERK, extracellular signal-regulated kinase; MAPK, mitogen-activated protein kinase; JNK1, c-Jun N-terminal kinase isoform 1.

To elucidate the downstream pathways through which the CCDC86-ATF3 axis modulated glioma development, GSEA was conducted on glioma samples from the METABRIC database, utilizing expression profiling data obtained from the cBioPortal (https://www.cbioportal.org/). Samples were stratified into high and low CCDC86 expression groups based on the median expression level of CCDC86. The analysis indicated activation of REACTOME_ACTIVATED_TAK1_MEDIATES_P38_MAPK_ACTIVATION (NES = 1.831; *p* = 0.0021), KEGG_GLYCOLYSIS_GLUCONEOGENESIS (NES = 1.66109; *p* = 0.0417), and WP_GLYCOLYSIS_AND_GLUCONEOGENESIS (NES = 1.5637; *p* = 0.0496) in the high CCDC86 expression group (Fig. 5A), implying a potential role for CCDC86 in regulating glioma glycolytic metabolism through the ERK/mitogen-activated protein kinase (MAPK) pathway. The ERK/MAPK pathway is known to play a crucial role in glioma progression, with its dysregulation linked to poor patient outcomes.^21^ Here, we observed a notable decrease in phosphorylated ERK, c-Jun N-terminal kinase isoform 1 (JNK1), and p38 levels in CCDC86-silenced U251 cells, while total protein levels remained unaffected (Fig. 5B). Subsequent data revealed that overexpression of CCDC86 in SHG-44 and U251 cell lines activated ERK/MAPK signaling, evidenced by increased phosphorylation of ERK, JNK1, and p38. These effects were reversed upon treatment with an ERK inhibitor, with no significant impact on the total protein levels of these factors (Fig. 5C). Moreover, CCDC86 overexpression promoted glioma cell proliferation while inhibiting apoptosis, partially reversed by ERK inhibitor treatment (Fig. 5D, E). To investigate whether CCDC86's regulation of ERK/MAPK signaling depended on ATF3, we assessed ERK and JNK1 levels and their phosphorylation status in SHG-44 and U251 cells with ATF3 knockdown, CCDC86 overexpression, or both. The results indicated that CCDC86 overexpression up-regulated p-ERK and p-JNK1, effects that were nullified after ATF3 silencing (Fig. 5F). Overall, CCDC86 might modulate the ERK signaling pathway through ATF3.

**Figure 6:**
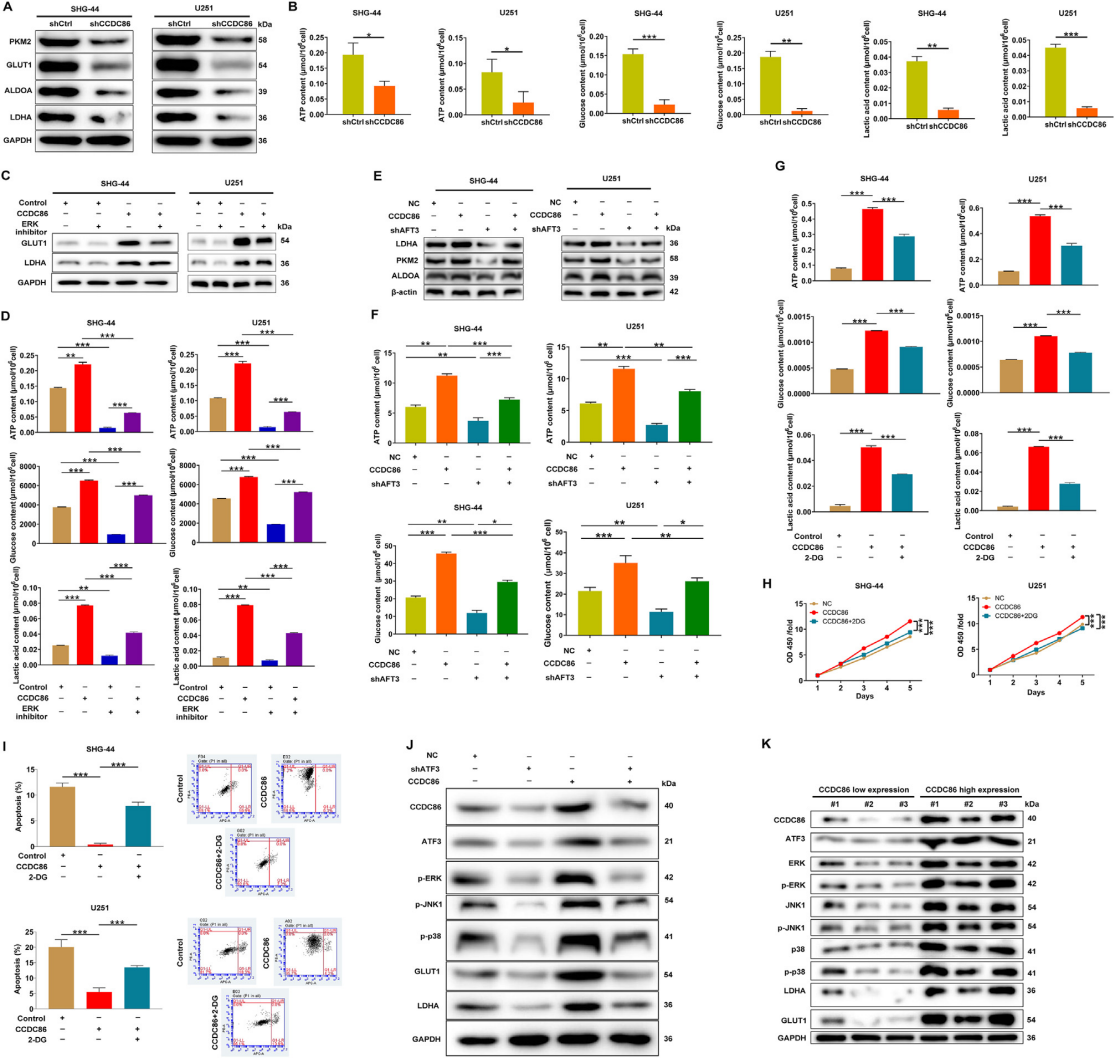
The CCDC86-ATF3 regulates glycolytic metabolism and tumor development. **(A)** Impact of CCDC86 knockdown on glycolysis-related protein levels in SHG-44 and U251 cells. **(B)** Effects of CCDC86 knockdown on ATP content, glucose uptake, and lactate production in SHG-44 and U251 cells. **(C)** Influences of the ERK pathway on glycolysis-associated protein expression in CCDC86-overexpressing cells. **(D)** Effects of ERK inhibitor on ATP content, glucose uptake, and lactate production in CCDC86-overexpressing cells. **(E)** Assessment of LDHA, PKM2, and ALDOA levels in SHG-44 and U251 cells with ATF3 knockdown, CCDC86 overexpression, and combined CCDC86 overexpression and ATF3 knockdown. **(F)** Assessment of ATP content and glucose uptake in SHG-44 and U251 cells with ATF3 knockdown, CCDC86 overexpression, and combined CCDC86 overexpression and ATF3 knockdown. **(G)** Assessment of glycolysis inhibition with 2-DG treatment in CCDC86-overexpressing cells. **(H, I)** Evaluation of cell proliferation (H) and apoptosis (I) following treatment with 2-DG in CCDC86-overexpressing cells. **(J)** Detection of ERK pathway-associated and glycolysis-related protein expression in tumor tissues with ATF3 knockdown, CCDC86 overexpression, and combined CCDC86 overexpression and ATF3 knockdown. **(K)** Patterns of CCDC86, ATF3, ERK pathway-associated, and glycolysis-related proteins in cancer tissues with high CCDC86 expression compared with adjacent normal tissues with low CCDC86 expression. NC (OE + KD): control; CCDC86+NC-shATF3: CCDC86 overexpression; shATF3+NC-CCDC86: ATF3 down-regulation; CCDC86+shATF3: CCDC86 overexpression and ATF3 down-regulation. ∗*p* < 0.05, ∗∗*p* < 0.01, and ∗∗∗*p* < 0.001. CCDC86, coiled-coil domain-containing 86; ATF3, activating transcription factor 3; ERK, extracellular signal-regulated kinase; LDHA, lactate dehydrogenase A; PKM2, pyruvate kinase M2; ALDOA, aldolase, fructose-bisphosphate A.

On the other hand, analysis of glycolysis-related proteins revealed a significant decrease in PKM2, glucose transporter type 1 (GLUT1), aldolase, fructose-bisphosphate A (ALDOA), and lactate dehydrogenase A (LDHA) levels in CCDC86-silenced SHG-44 and U251 cells (Fig. 6A). Knockdown of CCDC86 also resulted in decreased ATP content, glucose uptake, and lactate production in these cells (Fig. 6B). Subsequently, we explored the impacts of the ERK pathway on CCDC86-mediated glycolysis. Evaluation of glycolysis-associated protein expression in CCDC86-overexpressing cells via western blotting showed that an ERK inhibitor significantly reduced LDHA and GLUT1 protein levels (Fig. 6C). Forced expression of CCDC86 increased ATP content, glucose uptake, and lactate production, effects that were reversed by the ERK inhibitor (Fig. 6D). To further validate the role of ATF3 in CCDC86-mediated glycolysis, LDHA, PKM2, and ALDOA levels were assessed in glioma cells with ATF3 knockdown, CCDC86 overexpression, or both. Knockdown of ATF3 significantly reversed the enhanced effects of CCDC86 overexpression on these glycolytic protein levels (Fig. 6E). Corresponding glycolytic indicators such as ATP content and glucose concentration were also measured, with results confirming that knockdown of ATF3 significantly reversed the promoting effects of CCDC86 overexpression on glycolysis process (Fig. 6F). Further investigation using 2-DG (a glycolysis inhibitor) aimed to determine whether CCDC86 mediated tumor progression through the glycolysis process. Treatment with 2-DG significantly decreased ATP content, glucose uptake, and lactate production in CCDC86-overexpressing cells compared with untreated cells (Fig. 6G). Moreover, 2-DG treatment in CCDC86-overexpressing cells recapitulated the phenotypes, as evidenced by restored cell proliferation (Fig. 6H) and apoptosis (Fig. 6I). Additionally, *in vivo* experiments involved the detection of ERK pathway-associated and glycolysis-related proteins in tumor tissues. The results showed a significant increase in CCDC86, ATF3, and ERK pathway-associated and glycolysis-related protein expression in the CCDC86 overexpression group, while ATF3 knockdown led to their decrease. Also, tissues with both CCDC86 overexpression and ATF3 knockdown exhibited the reduction in the expression of these proteins compared with tissues with CCDC86 overexpression alone (Fig. 6J; Fig. S4C). Furthermore, ATF3 and ERK pathway-associated and glycolysis-related proteins were up-regulated in CCDC86 highly expressed cancer tissues compared with adjacent normal tissues with low CCDC86 expression (Fig. 6K). In summary, CCDC86 might promote glioma development by regulating the ERK pathway and glycolysis process.

## Discussion

Glioma presents a formidable obstacle in oncology, characterized by its aggressive behavior and the scarcity of viable treatment avenues, frequently resulting in poor patient prognoses. While progress has been made in unraveling the genetic underpinnings and signaling cascades implicated in glioma, the quest for efficacious therapies persists unfulfilled.

One promising avenue for therapeutic intervention is CCDC86, a previously unexplored potential target in glioma. Our study aimed to elucidate the role of CCDC86 in glioma pathogenesis. We provide evidence demonstrating that CCDC86 modulates the activity of the mitogen-activated protein kinase (MAPK)/extracellular signal-regulated kinase (ERK) pathway, a crucial signaling cascade implicated in glioma progression.^22^^,^^23^ The modulation of glycolytic metabolism by the ERK pathway was found to depend on CCDC86 expression levels, as indicated by the substantial decrease in GLUT1 and LDHA protein levels following ERK inhibition in CCDC86-overexpressing cells. Moreover, our findings revealed that CCDC86 overexpression promoted glioma cell proliferation while inhibiting apoptosis, effects that were partially reversed by treatment with an ERK inhibitor. These results highlighted the potential therapeutic relevance of targeting CCDC86 in glioma treatment strategies.

In this study, we explored the role of CCDC86 in glioma and its underlying mechanisms. Our results unveiled elevated expression levels of CCDC86 in glioma, correlating with unfavorable prognosis. The impact of CCDC86 in glioma was further defined with the identification of ATF3 as a downstream target gene, a transcription factor pivotal in regulating genes involved in cellular stress and cell cycle progression. ATF3, belonging to the ATF/CREB family,^24^ functions as a stress-responsive DNA-binding protein, orchestrating cellular responses to diverse stressors.^25^ It governs various cellular processes, including cell cycle regulation, apoptosis, differentiation, and immune responses, by binding to specific DNA sequences.^26^ In recent years, the role of ATF3 in cancer has been extensively studied, with conflicting reports suggesting its dual nature. While some research indicates ATF3's potential as a tumor suppressor, stifling cell proliferation and triggering apoptosis,^27^, ^28^, ^29^, ^30^ others propose its ability to foster tumor growth and metastasis.^31^, ^32^, ^33^ Our study demonstrated that down-regulating CCDC86 resulted in diminished ATF3 expression. Consequently, suppressing ATF3 impeded glioma cell proliferation and migration, counteracting the oncogenic effects induced by CCDC86 overexpression. Furthermore, we elucidated the involvement of ATF3 in the CCDC86-mediated ERK signaling pathway.

BHLHE40, a basic helix-loop-helix (bHLH) transcription factor, is known for its involvement in the regulation of cell differentiation, proliferation, and stress responses.^34^ In this study, co-immunoprecipitation experiments confirmed an interaction between BHLHE40 and CCDC86, suggesting a potential mechanism by which CCDC86 could influence ATF3 expression. Nuclear fractionation assays following CCDC86 knockdown indicated a decrease in BHLHE40 nuclear levels, implying that CCDC86 may be essential for the proper nuclear localization of BHLHE40. Chromatin immunoprecipitation assays further demonstrated that BHLHE40 could bind to the ATF3 promoter. The overexpression of CCDC86 resulted in increased enrichment of ATF3 promoter in BHLHE40-associated DNA complexes, suggesting that CCDC86 stabilizes ATF3 expression through its interaction with BHLHE40. These findings not only identify CCDC86 as a regulator of ATF3 but also highlight a novel molecular mechanism involving the transcription factor BHLHE40. The interaction between CCDC86 and BHLHE40 appears to be crucial for the transcriptional activation of ATF3, which may contribute to the oncogenic properties of glioma cells. By stabilizing ATF3 expression, CCDC86 potentially enhances the pro-tumorigenic signaling mediated by ATF3, offering a new perspective on the role of coiled-coil domain-containing proteins in cancer biology.

Glycolysis serves as a critical pathway in tumor cells, providing the primary energy source for their rapid expansion.^35^^,^^36^ Perturbations in the ERK signaling pathway significantly impact the glycolytic phenotype of gliomas. For example, phosphoglycerate kinase 1 (PGK1) plays a role in the development of renal clear cell carcinoma and resistance to sorafenib by activating the C-X-C motif chemokine receptor 4 (CXCR4)/ERK pathway, thereby accelerating glycolysis.^37^ Moreover, Rac family small GTPase 1 (Rac1) induces aldolase A and ERK signaling, leading to increased glycolysis.^38^ In our investigation, we found that elevated CCDC86 expression promoted glycolysis by up-regulating ATF3 and activating the ERK/MAPK pathway, thus driving glioma tumorigenesis.

In conclusion, our findings underscore the critical role of CCDC86 in promoting glioma progression. Targeting CCDC86 may emerge as a promising strategy for the development of novel glioma therapies.

## CRediT authorship contribution statement

**Jinping Liu:** Writing – original draft, Data curation. **Dingyu Du:** Writing – original draft, Formal analysis, Data curation. **Yukai Huang:** Writing – original draft, Formal analysis, Data curation. **Jie Tian:** Writing – review & editing, Software, Formal analysis. **Xuhui Wang:** Writing – review & editing, Conceptualization. **Longyi Chen:** Writing – review & editing, Conceptualization. **Feng Wang:** Writing – review & editing, Conceptualization.

## Funding

This study was conducted with support from the Key R&D Project of the Science and Technology Department of Sichuan Province, China (No. 2022YFS0146 to Longyi Chen).

## Conflict of interests

The authors declared no conflict of interests.
