# Supplementary material for: CCDC86-BHLHE40-ATF3 axis promotes aerobic glycolysis and tumor development in glioma
Source: Genes Dis. 2025 Apr 12;12(6):101643. doi: 10.1016/j.gendis.2025.101643 (PMC12361992; doi:10.1016/j.gendis.2025.101643)
Supplement: Multimedia component 1 [file mmc1.docx]

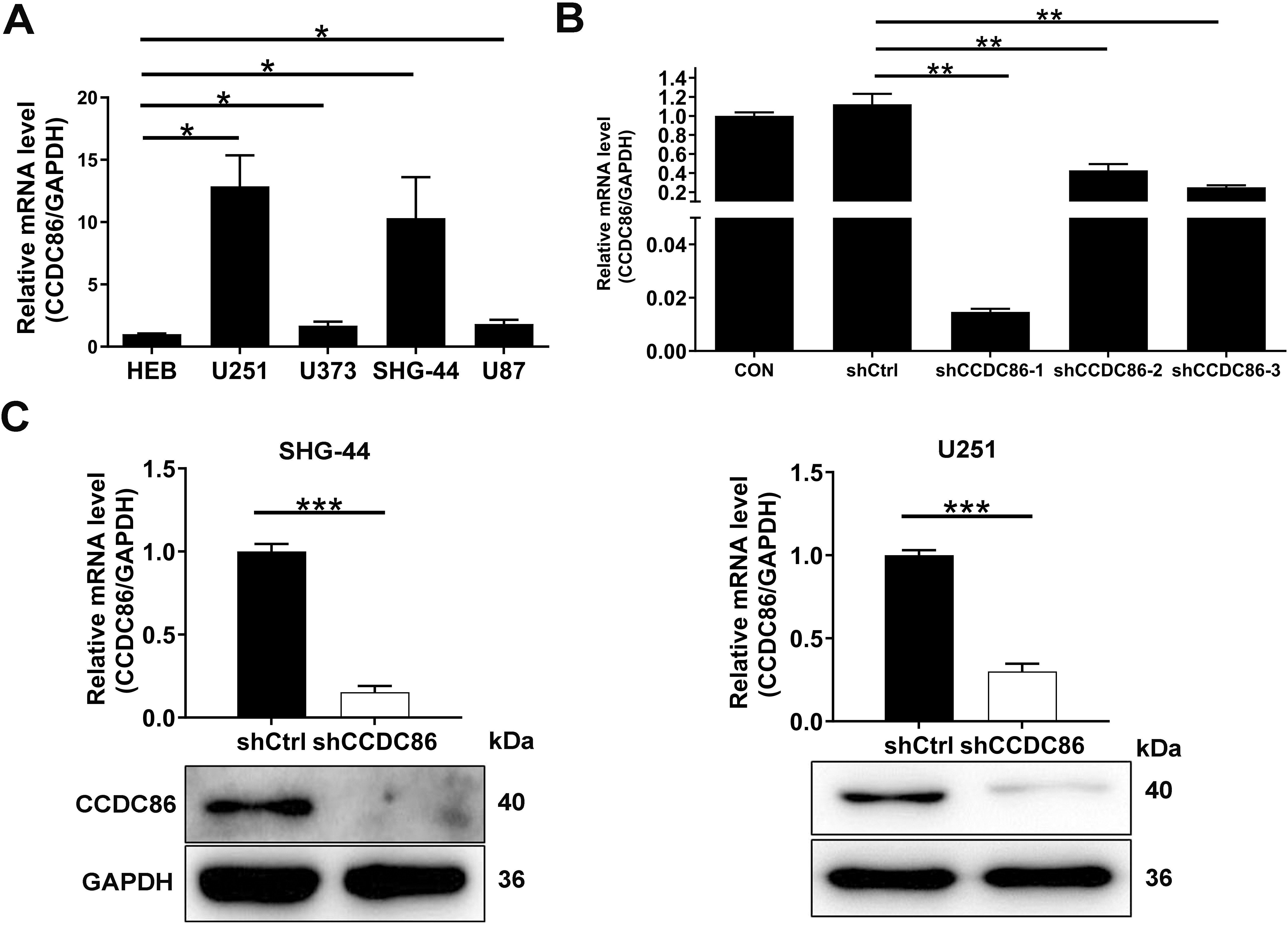


**Figure S1** **Endogenous CCDC86 levels and knockdown efficiency in glioma cell lines.** (A) Assessment of endogenous levels of CCDC86 in HEB and four glioma cell lines. (B) Evaluation of transfection efficiencies of shCCDC86-1, shCCDC86-2, and shCCDC86-3 via qRT-PCR in U251 cells. (C) Validation of CCDC86 knockdown in SHG-44 and U251 cells through qRT-PCR and western blot. * *P* < 0.05, ** *P* < 0.01, *** *P* < 0.001


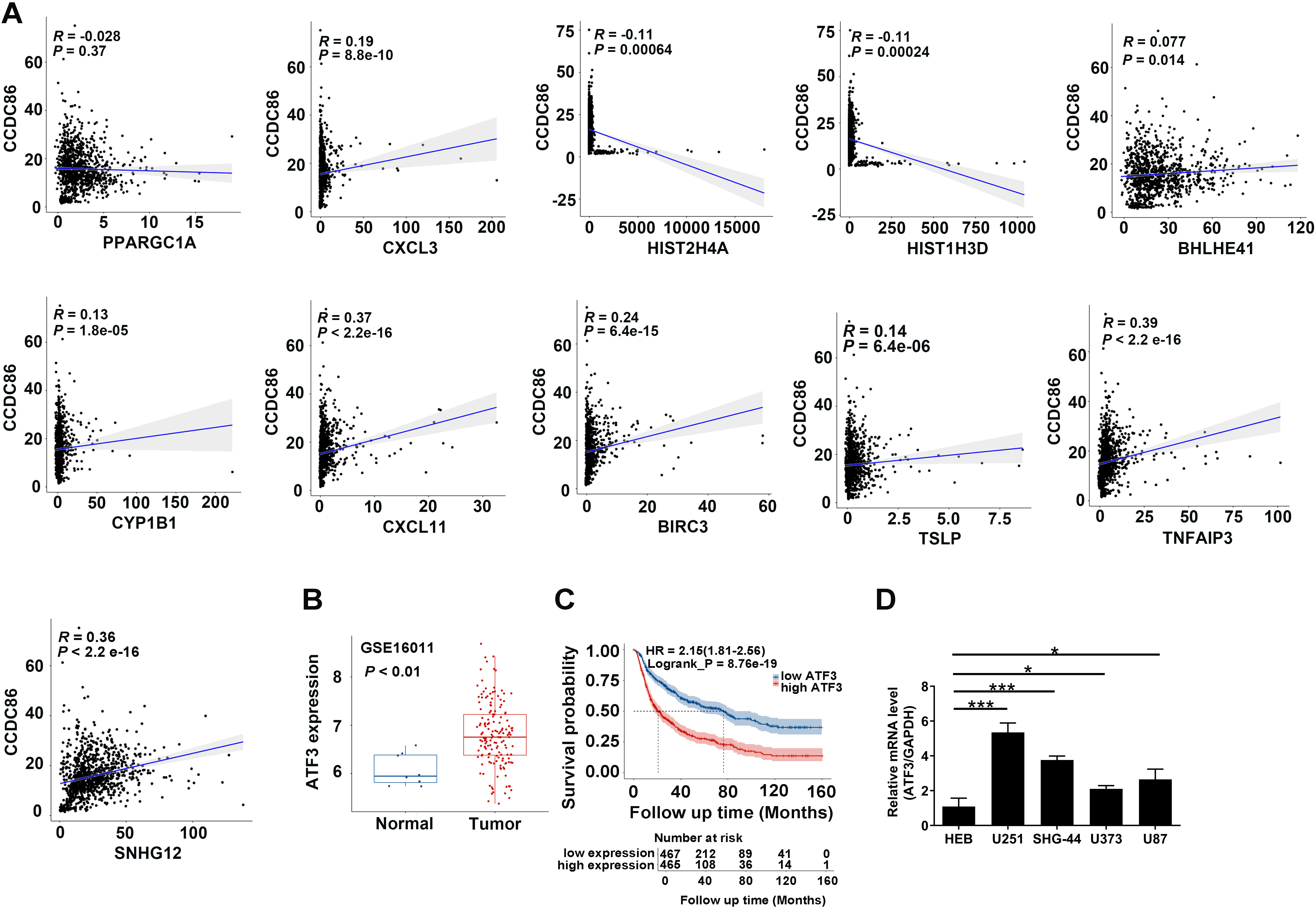


**Figure S2 CCDC86 correlations, ATF3 expression, and survival analysis in glioma.** (A) The correlation between CCDC86 with TNFAIP3, CXCL11, SNHG12, BIRC3, CXCL3, TSLP, CYP1B1, HIST1H3D, HIST2H4A, BHLHE4, and PPARGC1 based on the glioma samples from the CGGA database. (B) Differential Expression Analysis of ATF3 in glioma and normal tissues sourced from the GEO dataset GSE16011. (C) Log-rank test results showing a significant association between high ATF3 expression and poor overall survival in glioma patients. (D) Comparative assessment of endogenous ATF3 levels in HEB cells and four glioma cell lines. * *P* < 0.05, *** *P* < 0.001


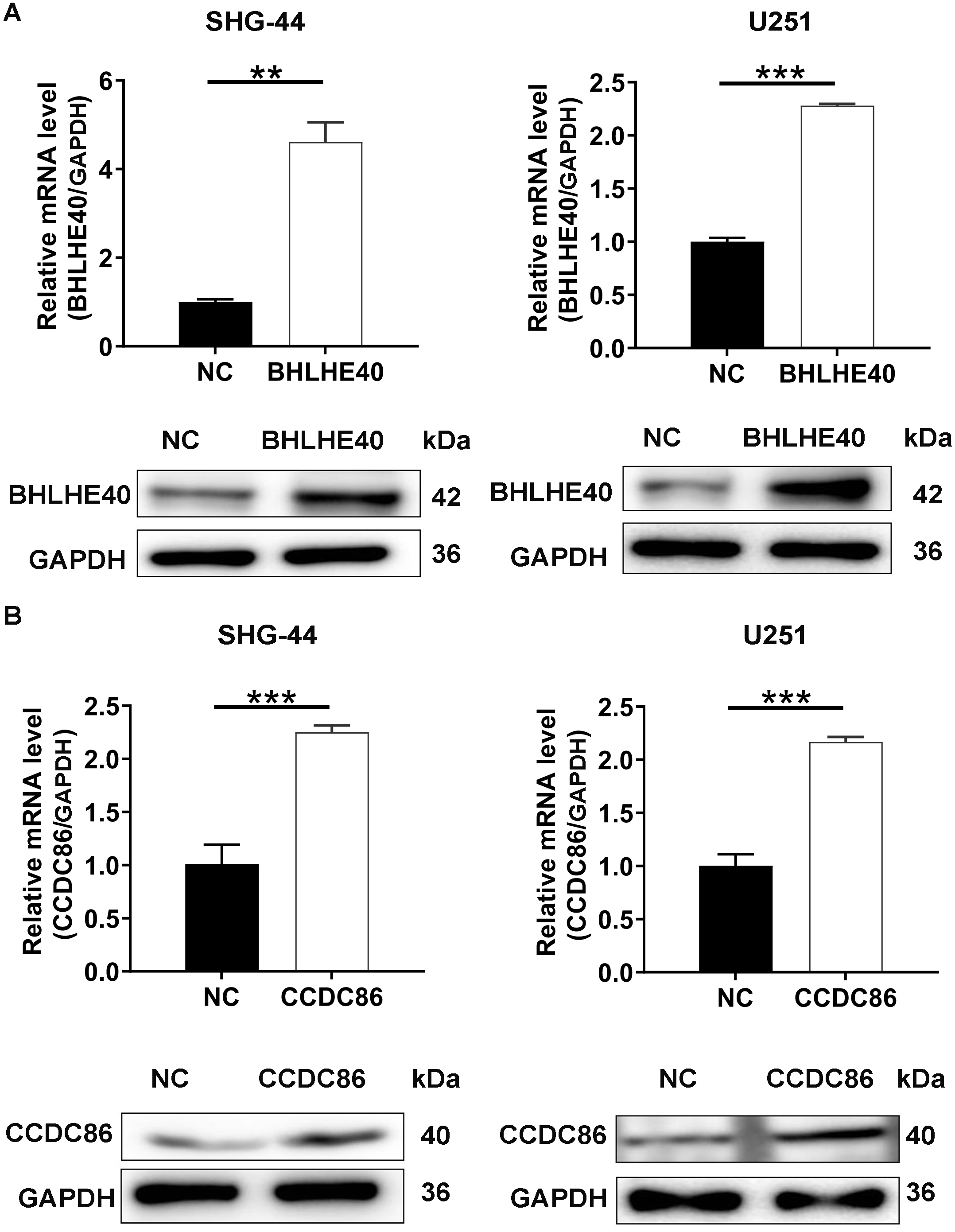


**Figure S3 Overexpression efficiency of BHLHE40 and CCDC86 in glioma cells.** (A, B) Confirmation of BHLHE40 overexpression (A) and CCDC86 overexpression (B) through qRT-PCR and western blot analysis in SHG-44 cells and U251 cells.


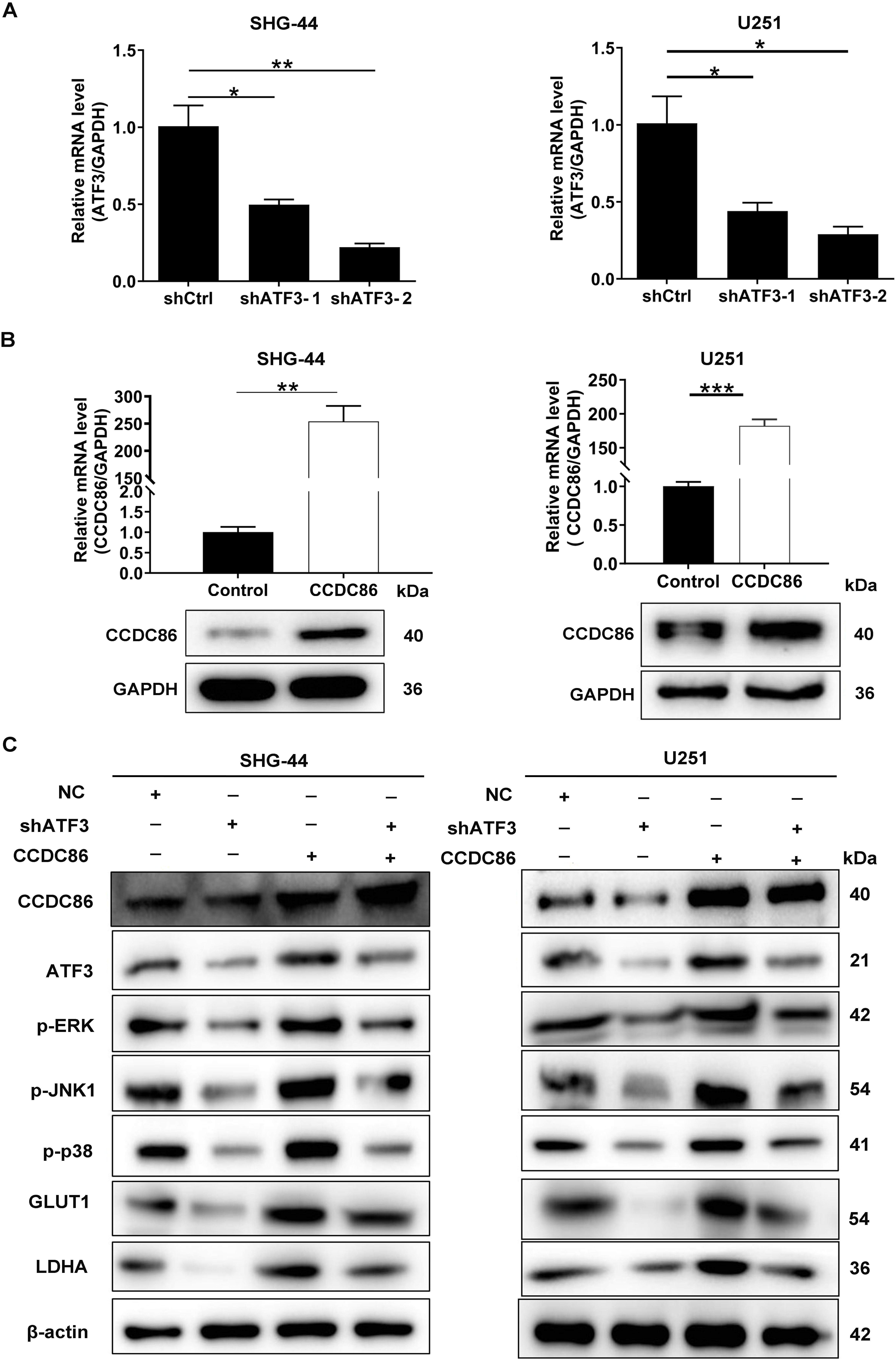


**Figure S4 ATF3 knockdown, CCDC86 overexpression, and their effects on ERK pathway and glycolysis in glioma cells.** (A, B) Confirmation of ATF3 knockdown (A) and CCDC86 overexpression (B) in SHG-44 cells and U251 cells. (C) Detection of ERK pathway-associated and glycolysis-related protein expression in tumor tissues with ATF3 knockdown, CCDC86 overexpression, and combined CCDC86 overexpression and ATF3 knockdown. NC (OE+KD): Control, CCDC86+NC-shATF3: CCDC86 overexpression, shATF3+NC-CCDC86: ATF3 downregulation, CCDC86+shATF3: CCDC86 overexpression and ATF3 downregulation. * *P* < 0.05, ** *P* < 0.01, *** *P* < 0.001

Table S1. The detail of antibodies in IHC staining and western blot.

| Primary antibodies | Dilution in IHC/WB | Source | | Company | Catalog No. |
| --- | --- | --- | --- | --- | --- |
| CCDC86 | 1:100 (IHC) | Rabbit | Proteintech | | 14947-1-AP |
| Ki67 | 1:100 | Rabbit | abcam | | ab16667 |
| CCDC86 | 1:1000 (WB) | Rabbit | Proteintech | | 14947-1-AP |
| ATF3 | 1:500 | Rabbit | Abcam | | ab216569 |
| ERK | 1:3000 | Rabbit | CST | | 4695 |
| p-ERK | 1:2000 | Rabbit | Proteintech | | 28733-1-AP |
| JNK1 | 1:3000 | Rabbit | Abcam | | ab179461 |
| p-JNK1 | 1:500 | Rabbit | Abcam | | ab131499 |
| p38 | 1:2000 | Rabbit | Proteintech | | 66234-1-Ig |
| p-p38 | 1:1000 | Rabbit | Proteintech | | 28796-1-AP |
| GLUT1 | 1:1000 | Rabbit | Proteintech | | 21829-1-AP |
| LDHA | 1:3000 | Rabbit | Abcam | | ab52488 |
| PKM2 | 1:5000 | Rabbit | Proteintech | | 15822-1-AP |
| ALDOA | 1:3000 | Rabbit | Proteintech | | 11217-1-AP |
| BHLHE40 | 1:2000 | Rabbit | Proteintech | | 17895-1-AP |
| Histone H3 | 1:3000 | Rabbit | Proteintech | | 17168-1-AP |
| GAPDH | 1:30000 | Mouse | Proteintech | | 60004-1-lg |
| β-Actin | 1:4000 | Mouse | Proteintech | | 66009-1-Ig |
| Secondary antibody | Dilution in IHC/WB |  | Company | | Catalog No. |
| Goat Anti-Rabbit | 1:3000 |  | Beyotime | | A0208 |
| Goat Anti- Mouse | 1:3000 |  | Beyotime | | A0216 |

Table S2. The primers in qRT-PCR and CHIP experiment.

| **Gene** | **Forward primer sequence (5′-3′)** | **Reverse primer sequence (5′-3′)** |
| --- | --- | --- |
| PPARGC1A | GGCAATAACTCCACCAAGAAAG | TCACCAAACAGCCGCAGACT |
| TNFAIP3 | CACGCTCAAGGAAACAGA | TTCCGAGTATCATAGCAAA |
| CXCL3 | CGCCCAAACCGAAGTCATA | GTGCTCCCCTTGTTCAGTATCT |
| HIST2H4A | AATGTGATTCGGGACGCAGT | ATCGAAACGTGCAAAGCTGG |
| HIST1H3D | CAACGACGAGGAGCTAAACAA | GCCATTGCGAACTTCTAAACC |
| ATF3 | AGGATTTTGCTAACCTGACGC | ACCTCGGCTTTTGTGATGG |
| BHLHE41 | CCTACAAATTACCGCACAGA | TTCTCCAGATGTCCCAGAGT |
| CYP1B1 | CGACCCCCAGTCTCAATCTC | GAGTCTCTTGGCGTCGTCAG |
| CXCL11 | TGTTCAAAAGAGGACGCTGTC | CTTGGGTACATTATGGAGGCT |
| BIRC3 | TGCTTTTGCTGTGATGGTGG | TGGCTTGAACTTGACGGATG |
| IFNB1 | ACGCCGCATTGACCATCTAT | GTCTCATTCCAGCCAGTGCTA |
| TSLP | CGTCGCTCGCCAAAGAAATGT | TGGTTGTGACTTTCCTTTTTCTCCT |
| SNHG12 | GTGATACTGAGGAGGTGAGCTTGTT | TTGCCTTCTGCTTCCCATAGAG |
| ATF3 promoter | GGAAGCCTCGGTGGGTTAGT | AGGCATGGTTGGTCAACTGAAG |
| GAPDH | TGACTTCAACAGCGACACCCA | CACCCTGTTGCTGTAGCCAAA |
